# The Photocatalytic and Antibacterial Performance of Nitrogen-Doped TiO_2_: Surface-Structure Dependence and Silver-Deposition Effect

**DOI:** 10.3390/nano10112261

**Published:** 2020-11-15

**Authors:** Abdul Wafi, Erzsébet Szabó-Bárdos, Ottó Horváth, Mihály Pósfai, Éva Makó, Tatjána Juzsakova, Orsolya Fónagy

**Affiliations:** 1 Department of General and Inorganic Chemistry, Center for Natural Sciences, University of Pannonia, H-8210 Veszprem, POB. 1158, Hungary; wafi@farmasi.uin-malang.ac.id (A.W.); bardose@almos.uni-pannon.hu (E.S.-B.); fonagyo@almos.uni-pannon.hu (O.F.); 2 Laboratory of Pharmaceutical Chemistry, Department of Pharmacy, Universitas Islam Negeri Maulana Malik Ibrahim Malang, Malang 65144, Indonesia; 3 Environmental Mineralogy Research Group, Research Institute of Biomolecular and Chemical Engineering, University of Pannonia, H-8210 Veszprem, POB. 1158, Hungary; posfaim@almos.uni-pannon.hu; 4 Department of Materials Engineering, Research Center for Engineering Sciences, University of Pannonia, H-8210 Veszprem, POB. 1158, Hungary; makoe@almos.uni-pannon.hu; 5 Laboratory for Surfaces and Nanostructures, Research Center for Biochemical, Environmental and Chemical Engineering, University of Pannonia, H-8210 Veszprem, POB. 1158, Hungary; yuzhakova@almos.uni-pannon.hu

**Keywords:** silver/nitrogen co-doped TiO_2_, visible-light-driven photocatalysis, hollow structure, coumarin, hydroquinone, disinfection

## Abstract

Catalysts for visible-light-driven oxidative cleaning processes and antibacterial applications (also in the dark) were developed. In order to extend the photoactivity of titanium dioxide into the visible region, nitrogen-doped TiO_2_ catalysts with hollow and non-hollow structures were synthesized by co-precipitation (NT-A) and sol–gel (NT-U) methods, respectively. To increase their photocatalytic and antibacterial efficiencies, various amounts of silver were successfully loaded on the surfaces of these catalysts by using a facile photo-deposition technique. Their physical and chemical properties were evaluated by using scanning electron microscopy (SEM), transmission electron microscopy–energy dispersive X-ray spectroscopy (TEM–EDS), Brunauer–Emmett–Teller (BET) surface area, X-ray diffraction (XRD), and diffuse reflectance spectra (DRS). The photocatalytic performances of the synthesized catalysts were examined in coumarin and 1,4-hydroquinone solutions. The results showed that the hollow structure of NT-A played an important role in obtaining high specific surface area and appreciable photoactivity. In addition, Ag-loading on the surface of non-hollow structured NT-U could double the photocatalytic performance with an optimum Ag concentration of 10^−6^ mol g^−1^, while a slight but monotonous decrease was caused in this respect for the hollow surface of NTA upon increasing Ag concentration. Comparing the catalysts with different structures regarding the photocatalytic performance, silverized non-hollow NT-U proved competitive with the hollow NT-A catalyst without Ag-loading for efficient visible-light-driven photocatalytic oxidative degradations. The former one, due to the silver nanoparticles on the catalyst surface, displayed an appreciable antibacterial activity, which was comparable to that of a reference material practically applied for disinfection in polymer coatings.

## 1. Introduction

Titanium dioxide (TiO_2_) is known as one of the most popular photoactive materials. It has emerged as an excellent photocatalyst for environmental applications due to its low cost, low toxicity, outstanding chemical stability, and unique photochemical properties. However, there are some properties which need to be improved for practical applications under visible light—e.g., the large band gap energy (∼3.2 eV for anatase) and fast recombination of photo-generated electron-hole pairs [1]. 

Up to now, many papers have reported different strategies in order to enhance the photoactivity of TiO_2_, such as impregnation with dye sensitizers and doping with nonmetal and metal elements. 

Dye sensitizers such as quinizarin and zinc protoporphyrin are commonly used to modify the TiO_2_ surface. These chromophore compounds are able to absorb visible light and excite electrons. The excited electrons then migrate to the conduction band of TiO_2_, leading to the formation of reactive oxygen species (ROS) [2,3,4].

On the other hand, doping with nonmetals, including N, F, S, C, and P, has been explored to extend the light absorption of TiO_2_ into the visible-light region. Nitrogen has been proven and considered as an effective dopant to narrow the band-gap energy due to its atomic size comparable to that of oxygen, high electronegativity and ionization energy, marked thermal stability, and cost-effectiveness [1,5].

Meanwhile, the incorporation of noble metals (such as Au, Ag, Pt, Cu, and Pd) onto the surface of N-TiO_2_ is also a favorable strategy to overcome the problem of fast recombination of the photo-generated electron-hole pairs and to improve the charge transfer [6,7,8,9]. Among them, Ag is the most suitable candidate for industrial applications due to its relatively low cost and easy preparation [10,11,12].

In addition, Ag nanoparticles also have been known to display strong cytotoxicity toward a wide range of bacteria, including *Escherichia coli* [13], *Staphylococcus aureus* [14], *Acinetobacter baumannii* [15], etc. The Ag nanoparticles can simply come into contact with the cell surfaces and destroy the membranes to inactivate bacteria. In another way, the inactivation process can be initiated by interaction of the bacteria with ROS (^•^O_2_^−^, ^•^OH) in the photocatalytic system [16,17]. Therefore, in addition to enhancing the photoactivity of N-TiO_2_, Ag doping is also expected to result in an antibacterial effect for selected microorganisms.

**Figure 1:**
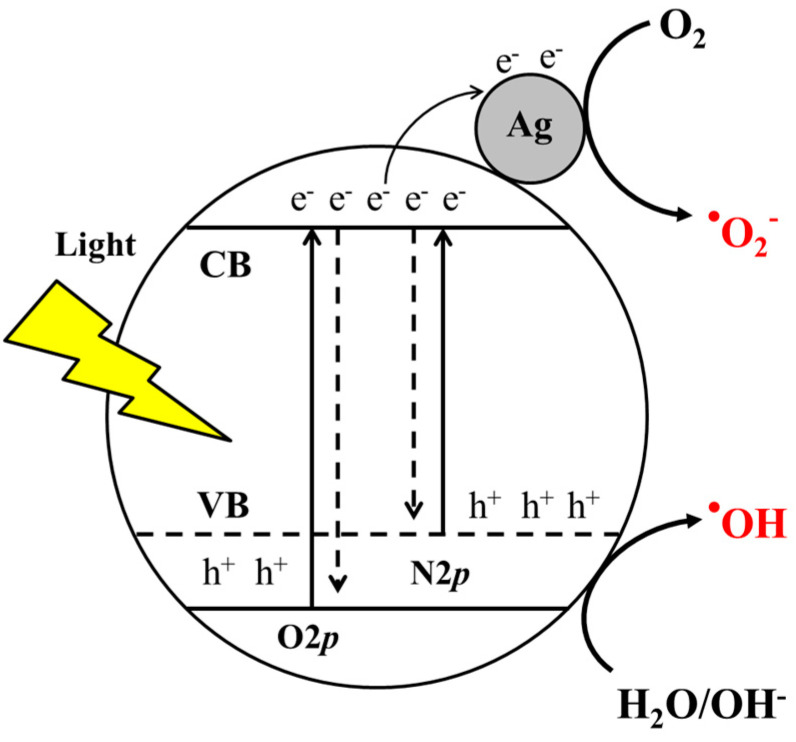
Photocatalytic reaction of Ag/N-TiO_2_.

Typically, when Ag/N-TiO_2_ catalysts are illuminated by light, electron-hole (e^−^, h^+^) pairs form, and then the interfacial charge transfers to Ag nanoparticles through the formation of Schottky barriers can suppress recombination of electrons and holes and extend their lifetimes in the Ag/N-TiO_2_ system. The surrounding O_2_ molecules can capture the electrons to form superoxide anion radicals (^•^O_2_^−^), and H_2_O molecules can be oxidized in the presence of holes to form hydroxyl radicals (^•^OH), as shown in Figure 1. These ROS (^•^O_2_^−^, ^•^OH) have strong oxidation potentials and can degrade numerous organic, frequently bio-resistant materials into harmless products [18,19,20]. 

Gao et al. prepared Ag/N-TiO_2_ via hydrothermal method with various Ag concentrations. The photocatalytic activity of an as-prepared sample was examined in Rhodamine B (RhB) solution under visible-light irradiation. It was found that the photocatalytic performance of Ag/N-TiO_2_ was affected by the concentration of Ag nanoparticles. It was reported that in the first period, the photocatalytic activity increased upon increasing Ag content, and then dropped above the optimal Ag content [10]. Gaidau et al. synthesized Ag/N-TiO_2_ grains by using electrochemical method. The photocatalytic experiment with the Orange II dye demonstrated that the photocatalytic activities of TiO_2_ under visible light could be improved by the synergistic effect of N doping and Ag modification [14]. Sun et al. successfully fabricated an Ag/N-TiO_2_ catalyst via an in situ calcination procedure, with titanium nitride (TiN) and silver nitrate (AgNO_3_) as starting materials. The catalyst displayed an enhanced light absorption and a red shift of the optical edge compared to pure TiO_2_ and N-TiO_2_. Under visible-light irradiation, a superior Methylene Blue (MB) degradation over Ag/N-TiO_2_ was also found compared to N-TiO_2_ [21]. However, these methods also involved special equipment or complex preparation processes. It is still necessary to develop more facile and efficient approaches. In addition, using dyes as organic model compounds could interfere with the photocatalytic performances of the catalysts studied due to their own light absorption and sensitizer properties. Therefore, other model compounds such as chemical probes or organic pollutants which do not absorb visible light must be utilized in the photocatalytic assessment of the catalysts.

In this work, N-TiO_2_ catalysts with hollow and non-hollow structures were fabricated via different methods such as co-precipitation and sol–gel procedures [22]. Titanium isobutoxide–urea (NT-U, sol–gel) and titanium isopropoxide–ammonium hydroxide (NT-A, co-precipitation) were used as catalyst precursors. In the designation of the catalysts, NT indicates N-doped TiO_2_, while U and A represent the nitrogen sources (urea and ammonium hydroxide).

Ag nanoparticles were also decorated on the surface of N-TiO_2_ via a facile photo-deposition method. Various concentrations of Ag were applied to investigate the photocatalytic efficiency under visible light. In addition, numerous measurements, including diffuse reflectance spectra (DRS), X-ray diffraction (XRD), scanning electron microscopy (SEM), transmission electron microscopy–energy dispersive X-ray spectroscopy (TEM–EDS), Brunauer–Emmett–Teller (BET) surface area, and inductively coupled plasma (ICP) spectroscopy were used for material characterization.

In order to assess the photocatalytic performances of the catalysts, coumarin was used as a chemical probe to analyze the formation of both ^•^OH and other reactive species (photo-generated electron or •O_2_^−^) under visible light [22]. Furthermore, to evaluate the performance of photocatalytic degradation regarding emerging contaminants in the environment, 1,4-hydroquinone (1,4-HQ) was also used as a model organic pollutant, which is a major benzene metabolite and commonly found in the industries of pharmaceutics and personal care [23,24]. Lastly, antibacterial effects of the catalysts were evaluated by using bioluminescence method in the presence of *Vibrio fischeri* strain. These bioluminescent bacteria are Gram-negative and commonly found in the marine environment [25].

## 2. Experimental

### 2.1. Materials

Titanium (IV) isobutoxide, (TTIB; Ti[OC(CH_3_)_3_]_4_, 98%), and titanium (IV) isopropoxide (TTIP; Ti[OCH(CH_3_)_2_]_4_, 98%), were purchased from Acros Organic (Geel, Belgium) and used as titanium precursors. Urea (CH_4_N_2_O) and ammonium hydroxide (NH_4_OH) 25% were used as nitrogen sources (pure reagent grade) and obtained from Scharlab Hungary Kft. (Debrecen, Hungary). Nitric acid (HNO_3_) 65% was supplied by VWR International Kft. (Debrecen, Hungary). Silver nitrate (AgNO_3_) and ethanol were purchased from Forr-Lab Kft. (Budapest, Hungary) and Molar Chemical Kft. (Halásztelek, Hungary). Coumarin (C_9_H_6_O_2_) and 7-hydroxycoumarin (C_9_H_6_O_3_, designated as 7-OHC) 99% were purchased from Carlo Erba Reagent (Cornaredo MI, Italy) and Sigma-Aldrich Kft. (Budapest, Hungary), respectively. 1,4-hydroquinone (C_6_H_6_O_2_) ≥99% was obtained from Sigma-Aldrich Kft. Compressed air was introduced (via bubbling) into the reaction mixtures from a gas bottle [22]. Freeze-dried bacteria (for Lumistox bacteria test) were provided by Hach Lange GmbH (Düsseldorf, Germany). 

### 2.2. Synthesis of N-doped TiO_2_

Sol–gel method (NT-U): A volume of 5 cm^3^ TTIB was dissolved drop-wise into 50 cm^3^ anhydrous ethanol. Furthermore, 3.6 g of urea in 2 cm^3^ of NH_4_OH was slowly added into the transparent solution under a vigorous stirring at room temperature for 2 h then temperature was increased to 80 °C for 1 h. Subsequently, a white gel was vacuum filtered and dried at 40 °C for 24 h [26]. 

Co-precipitation method (NT-A): The catalyst was prepared by using ammonium hydroxide as nitrogen precursor and the synthesis procedure was adopted from our previous work [22]. Typically, 85 cm^3^ of ammonium hydroxide solution (25%) was slowly added to 20 cm^3^ of nitric acid solution (65%), and then 50 cm^3^ of distilled water was also added to the transparent solution and stirred for 10 min. Subsequently, 2 cm^3^ of TTIP was incorporated into the solution and magnetically stirred for 60 min at 10 °C. Afterwards, the white precipitate formed was vacuum filtered and dried at 40 °C for 24 h. Finally, the N-TiO_2_ precursors obtained from these sol–gel and co-precipitation methods were ground and calcined at 450 °C for 30 min in an air atmosphere at a heating rate of 2 °C min^−1^ (in a Nabertherm P330 furnace, Nabertherm GmbH, Lilienthal, Germany). 

### 2.3. Synthesis of Ag/N Co-Doped TiO_2_

Ag nanoparticles were decorated on the surface of N-doped TiO_2_ by using photo-deposition method. Firstly, 0.180 cm^3^ solutions of various AgNO_3_ concentrations (0.2, 2.0, 20, and 200 mM) were diluted to 15 cm^3^ with distilled water. Then, 0.36 g of NT-U or NT-A was added into these solutions, followed by 10-min stirring to reach the adsorption-desorption equilibrium. Subsequently, under continuous stirring, the mixture was irradiated by using a UV LED (*λ*_max_ = 389 nm [22]) for 10 min from a distance of 5 cm. [27]. Lastly, the catalyst was dried at 50 °C for 24 h. The obtained catalysts are denoted as Ag/NT-U*x* and Ag/NT-A*x*, where *x* (*x* = 0, 10^−7^, 10^−6^, 10^−5^ and 10^−4^ mol g^−1^) represents the Ag/NT ratio. The color of the as-synthesized catalysts changed from light yellow to grey upon increasing the Ag concentration. 

In order to estimate the amount of Ag nanoparticles attached on the surface of the catalysts, the concentrations of Ag in the solution initially (i.e., before the adsorption process), after the adsorption, and at the end of the deposition were measured by using inductively coupled plasma optical emission spectroscopy (ICP-EOS, with a Spectroflame Modula equipment, SPECTRO Analytical Instruments, Kleve, Germany) under Ar plasma.

### 2.4. Characterizations

In order to identify the morphologies of the particles in the samples, an Apreo SEM (ThermoFisher Apreo S scanning electron microscope) was used at 5 kV for imaging. A Talos F200X G2 instrument (Thermo Fisher Scientific, Waltham, MA, USA), equipped with a field-emission gun and a four-detector Super-X energy-dispersive X-ray spectrometer was used at 200 kV for TEM and elemental analysis. High-resolution TEM (HRTEM) images were obtained for structure analysis, whereas scanning transmission electron microscopy (STEM) was used for obtaining high-angle annular dark-field (HAADF) images and EDS elemental maps. The specific surface area was determined by nitrogen adsorption/desorption isotherms measured with a Micromeritics ASAP 2000-type instrument on samples (weight ≈ 1.0 g) previously outgassed in vacuum at 160 °C. The surface areas of the samples were determined by the BET (Brunauer–Emmett–Teller) method from the corresponding nitrogen adsorption isotherms. The XRD patterns were obtained on a Philips PW 3710 type powder diffractometer (Philips Analytical, Almeao, Netherlands) with a Cu-Kα radiation source (*λ* = 1.5405 Å). Diffuse reflectance spectra (DRS) were recorded on a luminescence spectrometer (LS 50-B, PerkinElmer, Waltham, MA, USA) equipped with an integrating sphere attachment, and BaSO_4_ was used as a reference standard. The band-gap energy was calculated using Tauc plot of square of the Kubelka–Munk function against photon energy [22].

### 2.5. Photocatalytic Experiment

**Figure 2:**
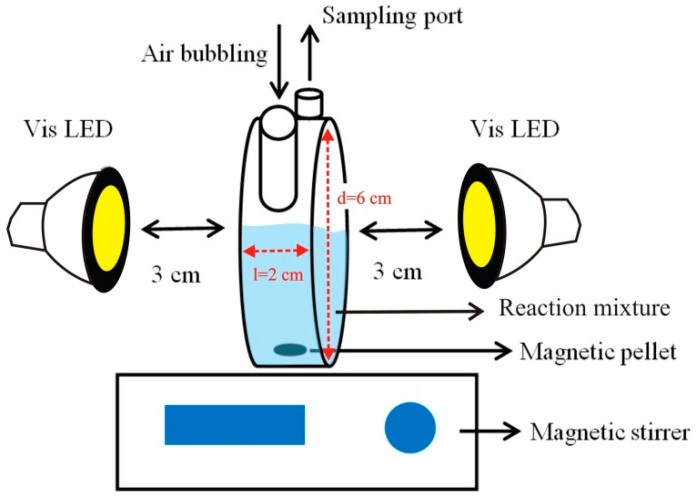
Schematic illustration of laboratory-scale quartz reactor, and its setup for photocatalytic experiments.

Photocatalytic experiments were carried out using a laboratory-scale quartz reactor with a volume of 50 cm^3^. Two visible LEDs (*λ*_max_ = 453 nm; 2 × 7 W) were used as light sources and located at both sides of the reactor with a distance of ≈ 3 cm, respectively. The lamp arrangement was modified and optimized from our previous work in order to reach a higher light intensity [22]. The optimum arrangement (light intensity = 90 mW cm^−2^ for each lamp) was obtained as illustrated in Figure 2. In all experiments, the reaction mixture was also stirred by air bubbling at a flow rate of 20 dm^3^ h^−1^. 

Typically, 50 mg of catalyst was added into 10 cm^3^ of distilled water and sonicated for 30 min to homogenize the particles. The suspension was left overnight under continuous stirring. Afterwards, 40 cm^3^ of coumarin (0.1 mM) or 1,4-hydroquinone (0.25 mM) was added into the suspension and left in the dark (30 min) to reach adsorption-desorption equilibrium. Then the visible LEDs were turned on. 

### 2.6. Analytical Procedure 

The samples were taken after given time intervals with a 5-cm^3^ syringe and filtered by a Millipore Millex-LCR PTFE 0.45 µm membrane filter. Furthermore, the absorbance of coumarin was measured by using a UV–Vis spectrophotometer (S-3100, Scinco, Seoul, Korea) and the emission of 7-OHC (*λ*_ex_ = 332 nm and *λ*_em_ = 453 nm) was recorded by a luminescence spectrometer (LS 50-B, PerkinElmer, Waltham, MA, USA) [22]. Luminescence method was also utilized for monitoring the photodegradation of 1,4-HQ due to its intense emission at *λ*_em_ = 330 nm (*λ*_ex_ = 288 nm). In addition, the concentration of 1,4-HQ was also analyzed by using a high performance liquid chromatograph (HPLC, Shimadzu, Kyoto, Japan) with a C18 column (Phenomenex Kinetec, 3.0 × 100 mm, 2.6 µm particle sizes, Phenomenex Inc., Torrance, CA, USA) for separation and a UV detector at 246 and 288 nm wavelengths. The mobile phase consisted of methanol and water (5/95%, *v*/*v*), and its flow rate was 0.2 cm^3^/min. The concentrations of 7-OHC, coumarin, 1,4-HQ were calculated from analytical standard curves. The mineralization process was measured by using a total organic carbon analyzer (TOC-L, Shimadzu, Kyoto, Japan).

### 2.7. Antibacterial Study 

The antibacterial effect was measured by using *Vibrio fischeri* luminescent bacteria. The sample preparation for antibacterial study is described in the Supplementary Information (SI) as Text S1 [28]. The luminescent intensity of *Vibrio fischeri* was detected by a Toxalert 100 device. The inhibition rate of bioluminescence could be achieved by Equation (1).

(1) $$Relative\;decomposition_{t}\left(\%\right)=\frac{I_{reference\left(t\right)}-I_{sample\left(t\right)}}{I_{reference\left(t\right)}}\times 100\;\;\;\;\;\;\;$$

where *I_reference(t)_* is the emission intensity of the reference or blind sample and *I_sample(t)_* is the emission intensity of the actual sample.

## 3. Result and Discussion 

### 3.1. Silver Deposition Analysis

**Figure 3:**
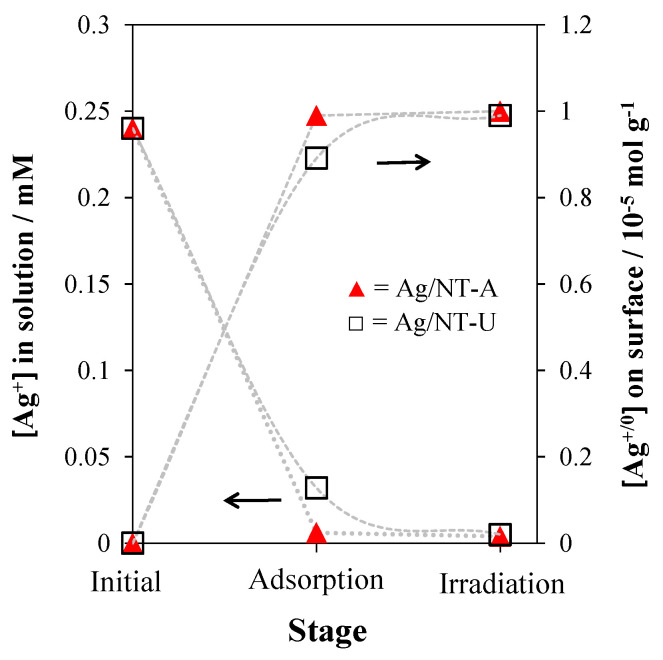
Change of Ag^+^ concentration in the solution phase (left *y* axis) and Ag^+^ (by adsorption) or Ag^0^ (by photo-reduction) concentrations on the catalysts (right *y* axis) during the adsorption and photo-deposition processes in the preparation of Ag/NT-A and Ag/NT-U catalysts (with 10^−5^ mol g^−1^ Ag concentration).

The Ag^+^ concentrations in each solution, initially (AgNO_3_ + water), after adsorption (AgNO_3_ + water + catalyst), and after UV irradiation (AgNO_3_ + water + catalyst + UV LED) were investigated by using ICP spectroscopy. Under dark conditions, a certain number of the dissolved Ag^+^ ions were adsorbed on the surfaces of the catalysts. NT-A adsorbed a higher amount of Ag^+^ compared to the case of the NT-U catalyst with concentrations of 0.97 × 10^−5^ and 0.89 × 10^−5^ mol Ag^+^ per g catalyst, respectively (Figure 3). However, after irradiation, all Ag^+^ ions (both on the catalyst surface and in the solution) were reduced to form Ag^0^ nanoparticles and deposited on the surfaces of NT-U and NT-A with concentrations of 0.99 × 10^−5^ and 1.00 × 10^−5^ mol g^−1^, respectively. 

Moreover, the adsorption efficiency depended on the initial Ag^+^ concentration in the solutions: 100%, 89%, and 79% of Ag^+^ were adsorbed from solutions of 0.024, 0.24, and 2.40 mM Ag^+^, respectively, on the surface of NT-U, resulting in 1.00 × 10^−6^, 0.89 × 10^−5^, and 0.79 × 10^−4^ mol g^−1^ concentrations. However, after irradiation, all Ag^+^ ions were successfully reduced and attached onto the surfaces of the catalysts. 

Typically, the photo-generated electrons on the surface of a catalyst are efficiently trapped by adsorbed Ag^+^ (rather than by oxygen), resulting in the formation of Ag^0^ nanoparticles. The holes then react with adsorbed water molecules to form oxygen and H^+^, according to the stoichiometry, as given in Equations (2) and (3) [29].

N-TiO_2_ {e^−^*,* h^+^} + Ag^+^_adsorbed_ → N-TiO_2_Ag^0^ {h^+^} (2)

(3) $$4{\mathrm{Ag}}^{+\;}+H_{2}O\underset{N-{\mathrm{TiO}}_{2}}{\to }\;4{\mathrm{Ag}}^{0\;}+4H^{+}+O_{2}$$

### 3.2. Structural and Elemental Analyses

**Figure 4:**
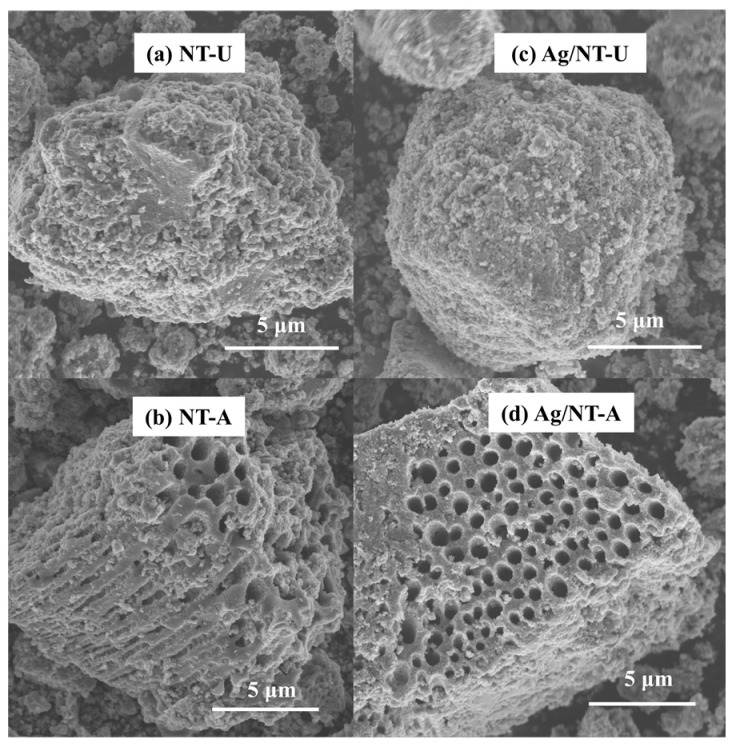
SEM morphologies of (**a**) NT-U, (**b**) NT-A, (**c**) Ag/NT-U, and (**d**) Ag/NT-A.

The morphologies and elemental compositions of the catalysts were studied by SEM and TEM–EDS measurements. Polydispersed (irregular) micro-particles were obtained for both NT-U and NT-A with sizes in the 1–50 µm range. SEM morphologies showed significant differences between NT-U and NT-A catalysts; the previous one possessed a non-hollow structure (Figure 4a), and the latter one exhibited a hollow structure (Figure 4b) [22]. Meanwhile, Ag deposition on the catalysts exhibited a negligible difference for the surfaces of Ag/NT-U and Ag/NT-A (Figure 4c,d). The diameters of the holes in the hollow structure (Ag/NT-A) were distributed in the 0.3–1.1 µm range, as shown in Figure S1. 

**Figure 5:**
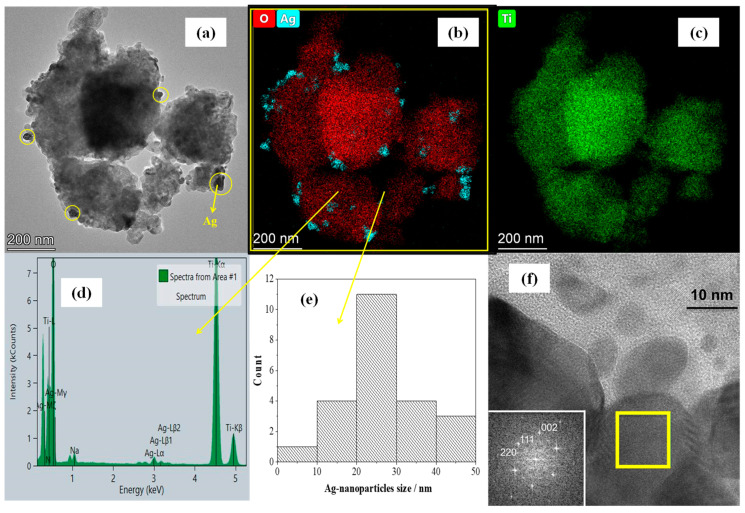
TEM analysis of the Ag/NT-U catalyst (with 10^−4^ mol g^−1^ Ag concentrtion); (**a**) TEM micrograph; (**b**,**c**) element maps obtained in STEM–EDS mode; (**d**) EDS spectrum of the entire aggregate; (**e**) size distribution of Ag nanoparticles; and (**f**) HRTEM image of Ag nanoparticles with an inserted fast Fourier transform (FFT) pattern on the lower left, obtained from the area marked by the yellow square and indexed according to the structure of native silver.

In TEM micrographs of Ag/NT-U 10^−4^, it is possible to observe the presence of quasi-spherical Ag nanoparticles on the catalyst’s surface (Figure 5a). The element maps and EDS spectra show that the catalyst consists of Ti and O as major elements. In addition, nanoparticles of silver as a dopant are unevenly distributed on the TiO_2_ aggregates (Figure 5b–d). The sizes of the Ag nanoparticles are typically in the range of 5 to 100 nm, but most of them are about 20 to 30 nm (Figure 5e). The TEM images and element maps for Ag/NT-A (with 10^−5^ mol g^−1^ concentration) and Ag/NT-U (with 10^−5^ and 10^−6^ mol g^−1^ Ag concentrations) are shown in Figure S2. Figure 5f shows an HRTEM image and the corresponding fast Fourier transform (FFT) pattern, suggesting that the nanoparticle consists of pure silver (Ag^0^).

The specific surface areas of the samples were measured by BET (Brunauer–Emmett–Teller) methods, as displayed in Table 1. The NT-A catalyst possessed a larger specific surface area (and pore volume) than NT-U did, with S_BET_ values of 61 and 32 m^2^ g^−1^, respectively. These values are in accordance with the different (hollow and non-hollow) structures shown by the SEM images (Figure 4). There are similar results in the literature. For instance, Suwannaruang et al. obtained about 34–42 m^2^ g^−1^ of specific surface area for N-TiO_2_ catalysts with nanorice structure prepared by using the hydrothermal method [30]. Their values indicate relatively even particle surfaces. 

Ag-loading on the NT-U enhanced the specific surface area to 47 m^2^ g^−1^, while it hardly changed in the case of NT-A (62 m^2^ g^−1^). These results may be interpreted by consideration of both the structures of the catalysts and their modification by the Ag nanoparticles deposited on the particles’ surfaces. The non-hollow structure of NT-U resulted in a lower specific surface area, which could be increased by the silver nanoparticles having considerably larger surfaces than the area they covered on the catalyst. The NT-A catalyst, however, possessed a significantly higher specific surface area due to the surficial holes with rather bent walls. Hence, deposition of Ag nanoparticles on these walls could not appreciably enhance the surface area; their own surface hardly exceeded the occupied area on the catalyst. A partly similar phenomenon was observed by Wang et al. regarding the specific surface areas of Ag/TiO_2_ nanofibers and nanotubes synthesized by general and emulsion electrospinning processes, respectively [20]. They reported that Ag-deposition on the TiO_2_ nanotubes enhanced the specific surface area from 60.58 to 76.93 m^2^ g^−1^. In contrast, the specific surface area of TiO_2_ nanofiber (53.17 m^2^ g^−1^) slightly decreased after Ag-loading (51.62 m^2^ g^−1^). Those results are also in accordance with the shapes of the catalyst surfaces.

**Figure 6:**
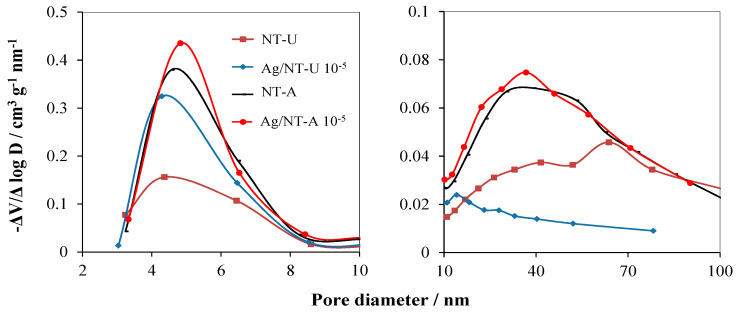
BJH pore-size distribution of the catalysts prepared.

The BJH (Barret–Joyner–Halenda) model was used to estimate the pore-size distribution of the samples in the range of 1.7–100 nm diameter. As shown in Figure 6, the surface of the NT-A catalyst possessed higher volumes of pores in the diameter range of 3–8.5 nm compared to those of NT-U. Ag-loading on the NT-U significantly enhanced the volumes of the pores in this diameter range, while it just slightly increased for the NT-A catalyst. Besides, much lower volumes of pores in the diameter range of 10–100 nm appeared for NT-A and Ag/NT-A catalysts, but still higher than for NT-U and Ag/NT-U. The higher volumes of pores with smaller diameters resulted in larger specific surface area of the catalysts, as indicated in Table 1. Besides, Figure 6 also suggests that silveration of NT-U resulted in the increase of the volumes of pores with smaller diameters by the decrease of volumes of pores with longer ones, partly covering the surfaces of larger pores.

**Figure 7:**
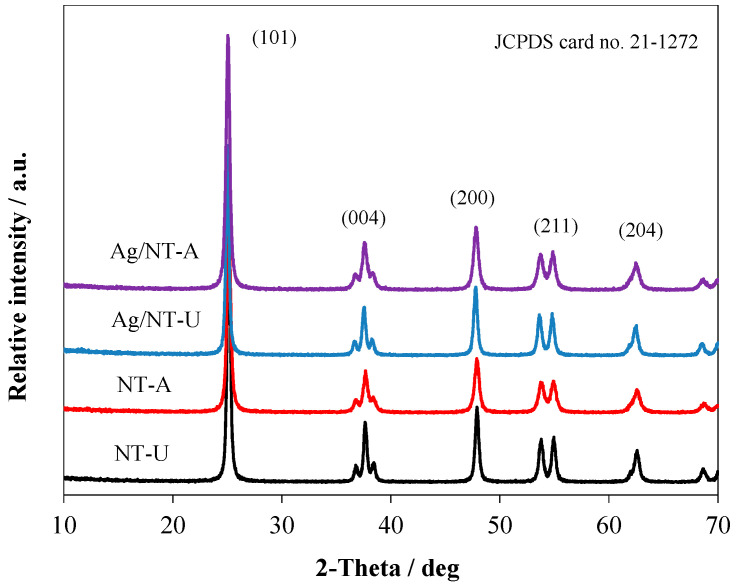
XRD patterns of the catalysts with 10^−5^ mol g^−1^ Ag concentration.

The XRD patterns of NT-U and NT-A catalysts are shown in Figure 7. Both catalysts displayed diffraction peaks located at 25.09°, 37.57°, 47.51°, 53.69°, and 62.43°, corresponding to (101), (004), (200), (211), and (204) crystal planes (respectively) of the anatase structure of TiO_2_ (JCPDS card number 21–1272) [22]. That clearly indicated that both NT-U and NT-A existed in pure anatase phase. The average of the crystallite size was calculated from broadening of XRD peaks by using the Scherrer equation. The NT-U catalyst displayed a higher crystallite size compared to that of NT-A, as shown in Table 1. The crystallite sizes of NT-U and NT-A were 25.2 and 19.0 nm, respectively, which implies that applications of different raw materials and preparation methods led to the formation of catalysts with identical crystalline phases, but different crystallite sizes.

Furthermore, no distinct silver signal was observed in the XRD spectra of Ag/NT-U and Ag/NT-A under various Ag loadings, as shown in Figure S3. It is highly likely that the low amount of Ag-loading remained below the detection limit of the equipment. As a result, all diffraction peaks of the silver-modified catalysts (Ag/NT-U and Ag/NT-A) are rather similar to the unmodified ones (NT-U and NT-A). In addition, silver-modification did not significantly affect the crystallinity of the catalysts either (Figure S3 and Table S1) [31,32]. Zhou et al. obtained a pure anatase phase for rod-like N-doped TiO_2_/Ag composites prepared by sol–gel method. The average crystallite size of the samples was 16.4 nm [33]. In addition, Gao et al. also reported a pure anatase phase of Ag/N-TiO_2_ prepared by hydrothermal method, with the average crystallite size of about 36.1 nm [10]. 

### 3.3. Band-Gap Energy 

**Figure 8:**
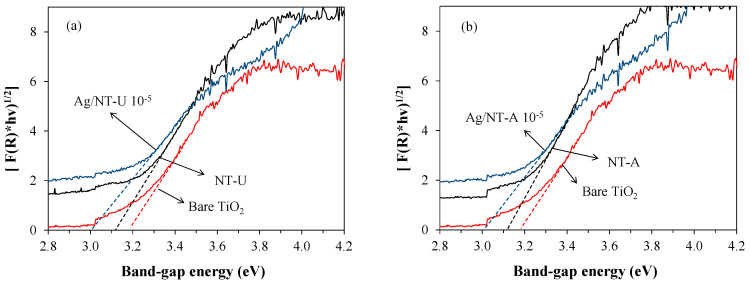
Effect of Ag-doping on the band-gap energies of (**a**) NT-U and (**b**) NT-A catalysts.

The optical properties of the catalysts were investigated by using DRS analysis. The band-gap energy was calculated from DRS spectra by application of the Kubelka–Munk function [22]. The summary of the band-gap energies of all samples is shown in Figure 8 and Table 1. Compared to bare TiO_2_ (3.18 eV), N-doping resulted in longer-wavelength absorption edge extending into the visible range, owing to a narrowed band-gap energy. In addition, Ag deposition also played a crucial role in the enhancement of light absorption, due to the surface plasmon resonance effect of Ag nanoparticles, which is owed to the high refractive index of the TiO_2_ in the surrounding medium [29,34,35,36]. The band-gap energies of catalysts with various silver-doping concentrations are shown in Table S1 and Figure S4.

### 3.4. Evaluation of Photoactivity by Application of Coumarin

Coumarin was used as a single probe in order to detect the formation of reactive species during the photocatalysis. Coumarin possibly reacts with ^•^OH and other reactive species to produce various hydroxylated coumarins (OHC, including 29% of 7-OHC [37]) and non-fluorescence products, respectively [15]. The concentration of coumarin from absorption spectra and that of 7-OHC from emission spectra were determined by using standard calibration curves [22]. 

**Figure 9:**
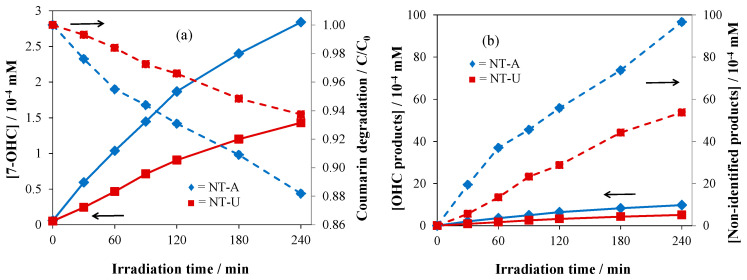
(**a**) 7-OHC formation vs. coumarin degradation and (**b**) formation of OHC vs. non-identified products.

As shown in Figure 9a, after 240-min irradiation time, the NT-U catalyst produced a lower concentration of 7-OHC (or ^•^OH) than NT-A did. The 7-OHC formations for NT-U and NT-A were 1.43 × 10^−4^ and 2.84 × 10^−4^ mM, respectively. This tendency was in agreement with the coumarin degradation, where NT-U and NT-A were able to degrade 6.26% and 11.82% of coumarin, respectively.

Furthermore, the amount of OHC was calculated from that of 7-OHC, according to coumarin degradation via ^•^OH reaction, and non-identified products were obtained from the difference between the amounts of totally degraded coumarin and formed OHC, representing the coumarin degradation through the reactions with other reactive species [22]. Figure 9b clearly shows that after 240 min irradiation, only 5.10 × 10^−4^ mM (NT-U) and 9.78 × 10^−4^ mM (NT-A) of coumarin reacted with ^•^OH. However, more coumarin reacted with other reactive species; 53.7 × 10^−4^ mM and 96.6 × 10^−4^ mM for NT-U and NT-A, respectively.

The better photocatalytic performance of NT-A might be due to its hollow structure. This feature is crucial in the photocatalytic performance of this catalyst, owing to its large surface area (61 m^2^ g^−1^) and thus more efficient adsorption of coumarin, compared to that of the non-hollow-structured NT-U (32 m^2^ g^−1^). Liu et al. reported that mesoporous nitrogen-doped TiO_2_ displayed a higher photocatalytic activity than the non-porous materials did under both UV and visible-light irradiations [38].

**Figure 10:**
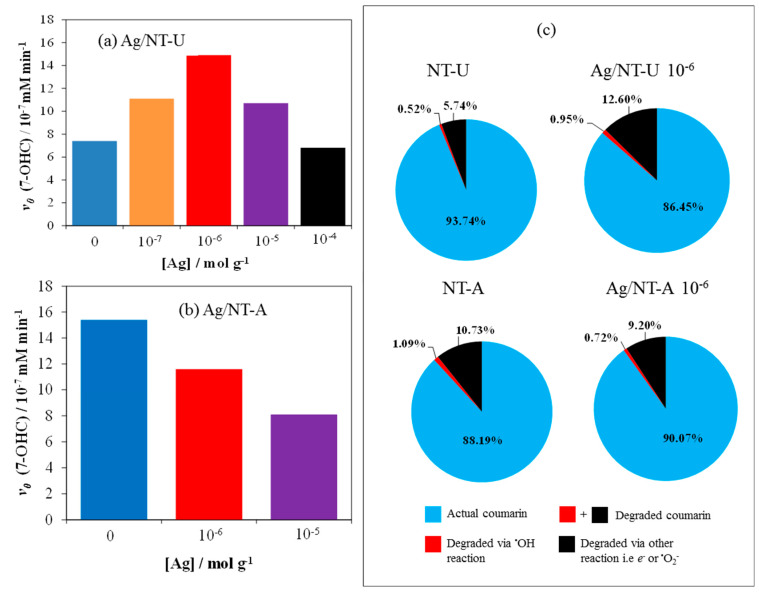
(**a**) Initial rate of 7-OHC formation on (**a**) Ag/NT-U and (**b**) Ag/NT-A catalysts, and (**c**) photocatalytic pathways on them after 240 min irradiation.

Besides specific surfaces areas, photocatalytic activities of Ag/N-TU and Ag/NT-A with various concentrations of Ag (as nanoparticles) were also investigated in coumarin solutions (Figure S5). The photocatalytic performances were evaluated on the basis of the initial rates of 7-OHC formation. Silver-modification on the surface of NT-U catalyst remarkably enhanced the formation of 7-OHC to the initial rate of 14.9 × 10^−7^ mM min^−1^ at the optimum Ag concentration of 10^−6^ mol g^−1^, as shown in Figure 10a. It is well known that silver-modification of such catalysts plays a crucial role in the photocatalytic activity, specifically trapping photo-generated electrons, and thereby promoting effective charge separations [27,39,40]. However, Ag-loading above 10^−6^ mol g^−1^ reduces the photocatalytic activity because too much silver on the catalyst surface could be detrimental to photonic efficiency. This phenomenon may be interpreted by consideration of several factors. As discussed above, silver nanoparticles can enhance the specific surface area of NT-U, which contributes to a better photocatalytic efficiency, along with increased charge separation. However, the coverage of the active excitable sites on the catalyst surface reduces the number of photons utilized for excitation of the semiconductor. Hence, these opposite effects led to a maximum efficiency at 10^−6^ mol g^−1^ silver concentration. The decrease of the active sites will be the dominant effect at higher Ag concentrations. Earlier literature also mentioned similar tendencies [34,41,42,43,44], but those studies dealt only with silveration of bare TiO_2_ catalysts (prepared by various methods) which were mostly applied for degradation of dyes or bacteria. In addition, Gao et al. also reported Ag-loading on the nitrogen-doped TiO_2_ catalyst via hydrothermal procedure [10]. In this case, however, compared to Ag/NT-U produced in our work, besides the different preparation method, a rather high Ag concentration (0.92 mol%) proved to be the optimum for photocatalytic degradation of RhB under visible light. This value is two orders of magnitude higher than 10^−6^ mol g^−1^, which corresponds to 0.008 mol%.

A significantly different tendency was observed with silver-modification on the surface of the hollow-structured NT-A catalyst (62 m^2^ g^−1^ specific surface area). A monotonous decrease in the photocatalytic activity was observed upon increasing the Ag concentration (to 10^−6^ and 10^−5^ mol g^−1^), compared to the case of the unmodified NT-A (Figure 10b). While no appreciable increase of the specific surface area was caused by the Ag-loading of NT-A (see in Table 1), the accessible active sites monotonously decreased in this case too. Therefore, the latter effect determined the results of silveration. 

Besides the formation of 7-OHC, transformation of coumarin into other hydroxylated derivatives and via reactions of other photogenerated reactants (such as *e*^−^ and ^•^O_2_^−^) was also determined (Figure 10c). In accordance with the results regarding 7-OHC formation, silver-loading of NT-U (with 10^−6^ mol g^−1^ Ag concentration) led to a significant increase of the transformation (degradation) in reactions with both ^•^OH (0.52–0.95%) and other reactive species (5.74–12.60%). In the case of NT-A, Ag-loading (at the same concentration) moderately decreased the formation of ^•^OH (1.09–0.72%) and other reactive species (10.73–9.20%) in agreement with Figure 10b. 

### 3.5. Evaluation of Photoactivity by Application of 1,4-hydroquinone

**Figure 11:**
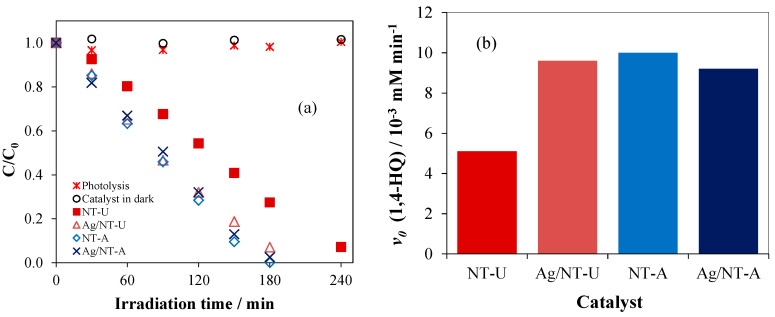
(**a**) C/C_0_ and (**b**) degradation rate of 1,4-HQ over various catalysts.

The photocatalytic efficiencies of the catalysts prepared were also investigated by the degradation of 1,4-HQ, using a method based on the luminescence of the starting compound (see Section 2.6). Additionally, blind probes (as comparisons) were measured: in the absence of catalyst (i.e., photolysis designated as “1,4-HQ + Vis”) and with catalyst in the dark (“1,4-HQ + NT-U”). In both blind probes, a negligible change of the initial concentration of 1,4-HQ was observed (Figure 11a). However, in the presence of catalysts, 1,4-HQ successfully decomposed to 100% after 180-min and 240-min irradiation times with NT-A and NT-U, respectively. In addition, the initial rates of photodegradation were 5.1 × 10^−3^ and 10 × 10^−3^ mM min^−1^ for NT-U and NT-A, respectively (Figure 11b). 

These results are in full agreement with those obtained for the degradation of coumarin. The same is valid for the observations with the silverized catalysts with 10^−6^ mol g^−1^ Ag concentration. Accordingly, the photocatalytic degradation of 1,4-HQ on Ag/NT-U was significantly more efficient than on the unmodified NT-U catalyst. In contrast, Ag-loading of NT-A slightly decreased the degradation efficiency. The comparisons of the rate data obtained for the unmodified and silverized catalysts are shown in Table 2. Besides the rates of 1,4-HQ degradation and their ratios (Ag/NT:NT), similar types of data are also shown regarding the reactions of coumarin with reactive species other than ^•^OH radical (i.e., *e*^−^ and ^•^O_2_^−^). The ratios of the corresponding rates measured under the same conditions agree well; 1.88 vs. 2.00 for Ag/NT-U vs. NT-U, and 0.92 vs. 0.91 for Ag/NT-A vs. NT-A, regarding 1,4-HQ vs. coumarin. These agreements indicate that, similarly to coumarin, hydroxylation is not the main degradation route for 1,4-HQ. This observation confirms our previous results [45], showing that the cleavage of the aromatic ring takes place via reactions other than hydroxylation, and it needs the presence of dissolved oxygen.

**Figure 12:**
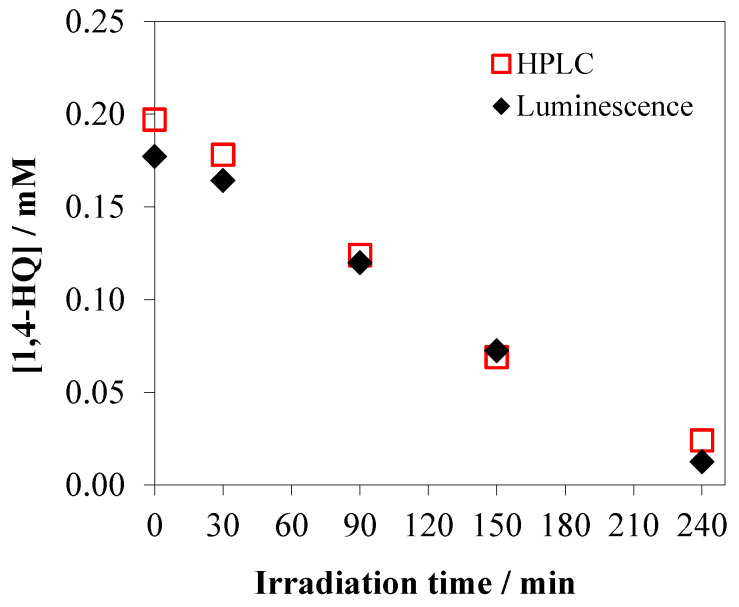
Comparison of HPLC and luminescence method for monitoring of photocatalytic 1,4-HQ degradation over NT-U catalyst.

HPLC analyses were also performed in order to investigate the degradation of 1,4-HQ on the NT-U catalyst. The concentrations of 1,4-HQ measured by using HPLC technique were compared to those obtained by the luminescence method. The results regarding the photocatalytic degradation of 1,4-HQ were in full agreement, as shown in Figure 12. This comparison confirmed the reliable applicability of the faster and simpler luminescence method. 

**Figure 13:**
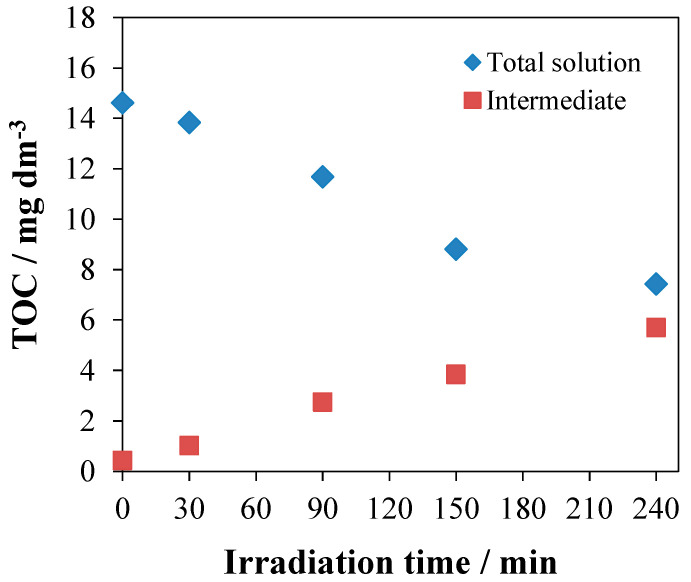
The change of the total organic carbon (TOC) values during the photocatalytic degradation of 1,4-HQ over NT-U catalyst.

TOC measurements were also carried out to clarify the mineralization process. The TOC representing the intermediates was estimated from the difference between the total TOC concentration of the reaction mixture and that of the unreacted 1,4-HQ. The result indicated that the TOC concentration of intermediate products increased during the photodegradation, while the total TOC of the reaction mixture steadily dropped from 14.6 to 7.4 mg dm^−3^ (Figure 13). It implies that a considerable part of the intermediates was mineralized to CO_2_ and H_2_O. 

The intermediates formed from 1,4-HQ are mostly short-chain acids, as observed earlier in similar systems [24,46,47,48,49]. Generally, the produced reactive oxygen species attack the phenyl ring of 1,4-HQ, producing dihydroxy derivatives (via ^•^OH reaction) or promoting aromatic ring cleavage (via ^•^O_2_^−^ reaction). The mineralization of the intermediates, according to the TOC results, along with our earlier observation [45] that ^•^OH alone cannot cleave aromatic rings, confirm that other reactive photogenerated species (*e*^−^ and ^•^O_2_^−^) play crucial roles in the degradation of these aromatic compounds.

### 3.6. Antibacterial Effect

The antibacterial effects of the catalysts were studied using *Vibrio fisheri* bacteria, as described in the SI (Text S1). The catalysts were fixed in an acrylate-based polymer on the surface of plastic sheets. The toxicity effects were measured by inhibition of the bioluminescence intensity of the bacterial suspension in contact with the catalysts (Figure S6, Tables S2 and S3). A commercially available plastic sheet with antibacterial surface was used as a control sample for comparison. Table 3 indicates that silver doping on both NT-U and NT-A could enhance the toxicity effect compared to the unmodified catalysts. The effect of Ag could be attributed to the fact that when Ag nanoparticles interact with microorganisms such as bacteria, silver ions (Ag^+^) are released and damage these organisms by attacking the negatively-charged cell walls, thereby deactivating cellular enzymes and disrupting membrane permeability; accordingly, cell lysis and cell death occur [50,51,52]. The maximum effects were observed at 10^−6^ mol g^−1^ Ag concentration for both Ag/NT-U and Ag/NT-A, with the values of 98% and 61.2%, respectively (Table 3). 

## 4. Conclusions

In this study, visible-light-active N-TiO_2_ photocatalysts were successfully synthesized through sol–gel (NT-U) and co-precipitation (NT-A) methods. The two differently prepared N-TiO_2_ catalysts exhibited different morphologies (hollow NT-A and non-hollow NT-U) and photoactivities in both coumarin and 1,4-HQ solutions. A facile photo-deposition method was used to decorate N-TiO_2_ surfaces with Ag nanoparticles. The Ag concentration played a critical role in the photoactivity of Ag/N-TiO_2_. The optimum Ag concentration (as low as 10^−6^ mol g^−1^) doubled the photocatalytic efficiency of non-hollow NT-U. However, Ag-loading on the hollow surface of NTA was not favorable for photocatalytic enhancement. Comparing the catalysts with different structures regarding the photocatalytic performance, silverized non-hollow NT-U proved competitive with the hollow NT-A catalyst without Ag-loading. Since the purpose of silveration was also to make these catalysts efficiently antibacterial, Ag-NT-U with 10^−6^ mol g^−1^ Ag concentration proved to be optimal, considering both photocatalytic and disinfectional activities.

## Figures and Tables

**Table 1:** Characteristic values of the catalysts prepared.

Catalyst	S_BET_ Values/m^2^ g^−1^	Pore Volume V_1.7__–__100 nm_/cm^3^ g^−1^	Crystallite Size/nm	Band-Gap Energy/eV
NT-U	32	0.08456	25.2	3.11
Ag/NT-U 10^−5^	47	0.10149	24.8	3.01
NT-A	61	0.14307	19.0	3.13
Ag/NT-A 10^−5^	62	0.14563	18.8	3.01

V_1.7–100 nm_—BJH cumulative desorption pore volume of pores with diameters between 1.7 and 100 nm.

**Table 2:** Ratios (Ag/NT: NT) of 1,4-HQ degradation and coumarin reaction with other reactive species.

Catalyst	*v_0_* (1,4-HQ) /10^−3^ mM min^−1^	Ratio	*v_0_* (Other Reactive Species) /10^−3^ mM min^−1^	Ratio
NT-U	5.10	1.88	2.33	2.00
Ag/NT-U 10^−6^	9.60		4.68	
NT-A	10.00	0.92	3.79	0.91
Ag/NT-A 10^−6^	9.20		3.44	

**Table 3:** Antibacterial effects of various catalysts compared to the control sample after 90-min contact.

Ag-Loading/mol g^−1^	Ag/NT-U/%	Ag/NT-A/%
0	40.4	30.0
10^−6^	98.0	61.2
10^−5^	70.0	52.2
10^−4^	46.8	40.5

## Supplementary Materials

The following are available online at https://www.mdpi.com/2079-4991/10/11/2261/s1. Text S1. Sample preparation for antibacterial study. Figure S1: Hollow-diameter distribution of Ag/NT-A. Figure S2: HAADF-TEM images and element maps of (a) Ag/NT-A 10−5, (b) Ag/NT-U 10−5, and (c) Ag/NT-U 10−6 (10−5 and 10−6 are the Ag concentrations in mol g−1). Figure S3: XRD patterns of (a) Ag/NT-U and (b) Ag/NT-A with different Ag-loadings. Table S1: Crystallite sizes and band-gap energies of Ag/NT-U and Ag/NT-A with different Ag-loadings. Figure S4: Band-gap energies of (a) Ag/NT-U and (b) Ag/NT-A with different Ag-loadings. Figure S5:(a,b) 7-OHC formation and (c,d) coumarin degradation over Ag/NTU and Ag/NT-A with different Ag-loadings. Figure S6: Luminescence intensity of bacterial suspension in the presence of (a) Ag/NT-U and (b) Ag/NT-A catalysts (with 10−6 mol g−1 Ag concentration) compared to the reference and control samples. Table S2: Relative decomposition of bacteria in the presence of NT-U and Ag/NT-U (with 10−6 mol g−1 Ag concentration) catalysts and the control sample. Table S3: Relative decomposition of bacteria in the presence of NT-A and Ag/NT-A (with 10−6 mol g−1 Ag concentration) catalysts and the control sample.
