# Supplementary material for: The Photocatalytic and Antibacterial Performance of Nitrogen-Doped TiO2: Surface-Structure Dependence and Silver-Deposition Effect
Source: Nanomaterials (Basel). 2020 Nov 15;10(11):2261. doi: 10.3390/nano10112261 (PMC7697533; doi:10.3390/nano10112261)
Supplement: Supplementary file 1 [file nanomaterials-10-02261-s001.pdf]

Supplementary Information

for

Photocatalytic and Antibacterial Performance of Nitrogen-doped TiO_2: Surface-Structure Dependence and Silver-Deposition Effect

Abdul Wafi[1,5], Erzsébet Szabó-Bárdos[1], Ottó Horváth[1*], Mihály Pósfai[2], Éva Makó[3], Tatjána Juzsakova[4], Orsolya Fónagy[1]

[1] Department of General and Inorganic Chemistry, Center for Natural Sciences, University of Pannonia, H-8210 Veszprem, POB. 1158, Hungary; wafi@farmasi.uin-malang.ac.id (A. W.); bardose@almos.uni-pannon.hu (E. S.-B.); fonagyo@almos.uni-pannon.hu (O. F.)

[2] Environmental Mineralogy Research Group, Research Institute of Biomolecular and Chemical Engineering, University of Pannonia, H-8210 Veszprem, POB. 1158, Hungary; posfaim@almos.uni- pannon.hu (M. P.)

[3] Department of Materials Engineering, Research Center for Engineering Sciences, University of Pannonia, H-8210 Veszprem, POB. 1158, Hungary; makoe@almos.uni-pannon.hu (É. M.)

[4] Laboratory for Surfaces and Nanostructures, Research Center for Biochemical, Environmental and Chemical Engineering, University of Pannonia, H-8210 Veszprem, POB. 1158, Hungary; yuzhakova@almos.uni-pannon.hu (T. Y.)

[5] Laboratory of Pharmaceutical Chemistry, Department of Pharmacy, Universitas Islam Negeri Maulana Malik Ibrahim Malang, 65144 Malang, Indonesia.

* Correspondence: horvath.otto@mk.uni-pannon.hu (O. H.);

Content **Page Nr.**

Text S1, Figure S1	2
Figures S2	3
Figure S3, Table S1, Figure S4	4
Figures S5	5
Figure S6, Tables S2-3	6

Text S1. Sample preparation for antibacterial study

For measurements of the antibacterial effect, the catalyst samples were immobilized in an acrylate based polymer on a plastic surface. The catalyst concentration on the plastic surface was 485 mg/m^2. Discs of 1 cm diameter were cut from this plastic sheet and fixed to the bottom of small, open sample containers (glass tubes) with screw caps. The bacterial suspensions were layered on the samples. The antibacterial effects of the catalysts were measured by using *Vibrio fischeri* luminescent bacteria. After reconstitution, the guaranteed life-span of bacteria was 4 hours. The suspension of bacteria was incubated for 80 minutes at 15°C. At the end of the incubation period, 0.5 cm^3 sample was measured into the sample holders, which were kept in the dark during the experiment, and the emission intensities of the samples were recorded at defined times. The reference sample was stored in a bottle, which did not contain any plastic disc. The control sample contained a disc from a commercially available plastic sheet with antibacterial surface. During the evaluation, the results obtained from 3 parallel measurements were averaged and then the relative decomposition percentage was calculated. Afterwards, the relative decomposition (%) of the actual sample was compared to the control one.

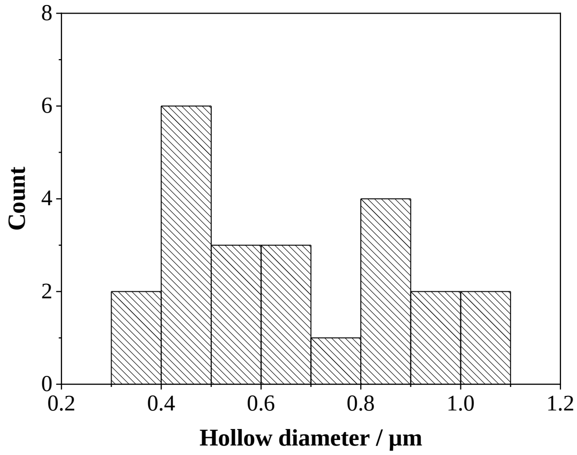

Figure S1. Hollow-diameter distribution of Ag/NT-A.

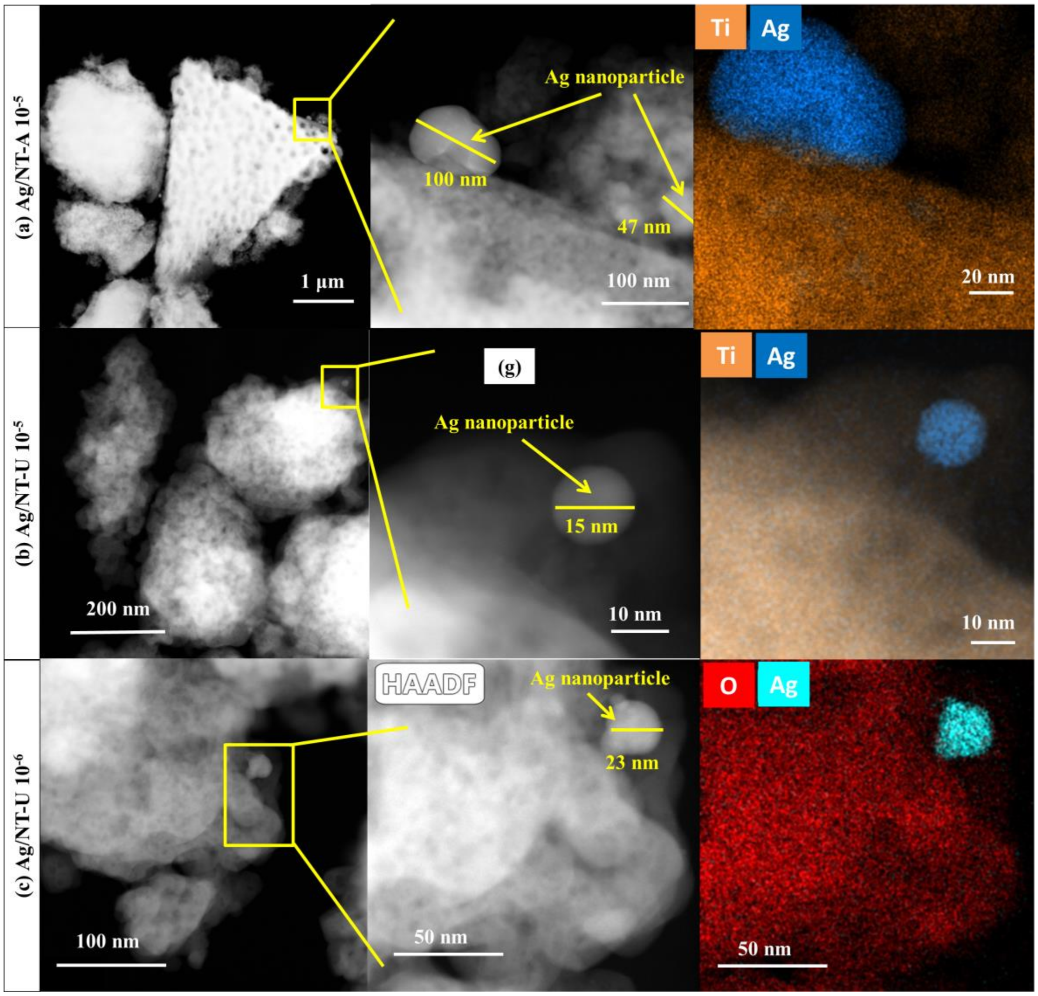

Figure S2. HAADF-TEM images and element maps of (a) Ag/NT-A 10^{-5}, (b) Ag/NT-U 10^{-5} and (c) Ag/NT-U 10^{-6} (10^{-5} and 10^{-6} are the Ag concentrations in mol g^{-1}).

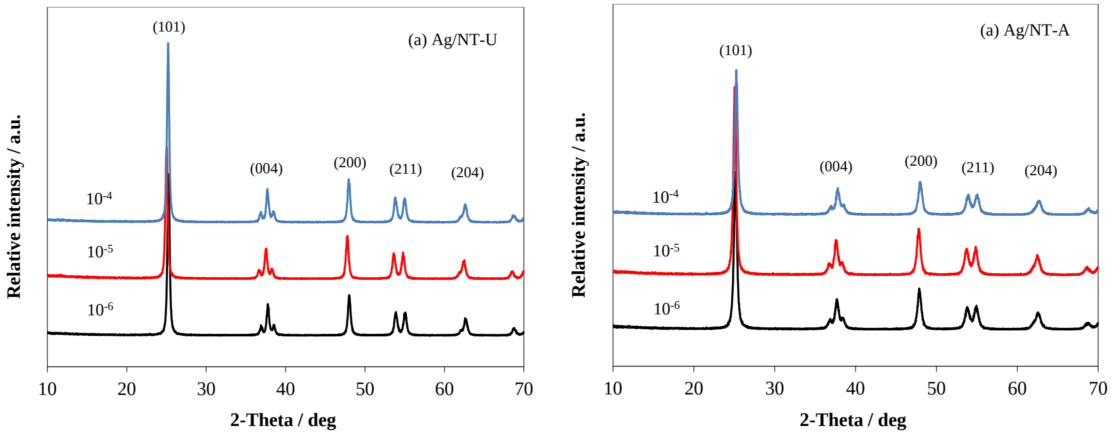

Figure S3. XRD patterns of (a) Ag/NT-U and (b) Ag/NT-A with different Ag-loadings.

Table S1. Crystallite size and band-gap energy of Ag/NT-U and Ag/NT-A with different Ag-loadings.

Catalyst	Ag content / mol g^{-1}	Crystallite size / nm	Band-gap energy / eV
Ag/NT-U	10^{-6}	25.2	3.07
	10^{-5}	24.8	3.01
	10^{-4}	25.4	2.96
Ag/NT-A	10^{-6}	17.3	3.06
	10^{-5}	18.8	3.01
	10^{-4}	17.4	2.98

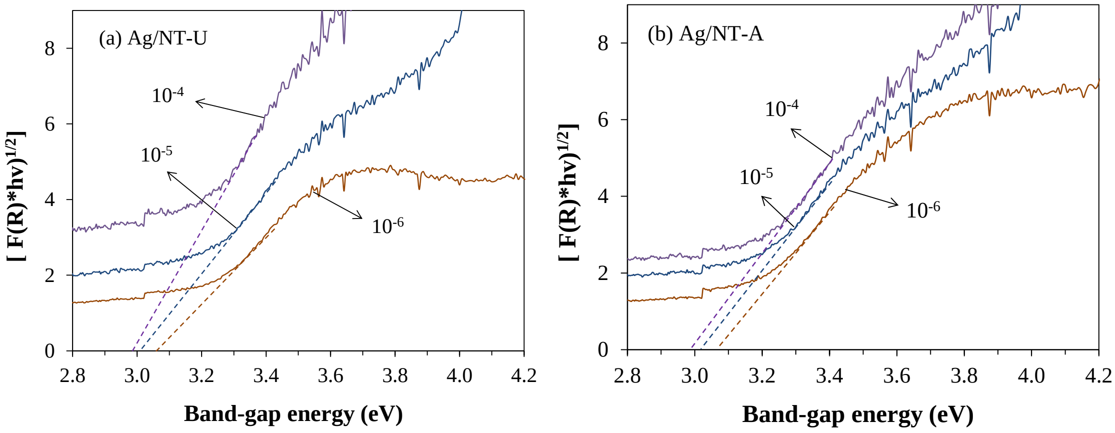

Figure S4. Band-gap energy of (a) Ag/NT-U and (b) Ag/NT-A with different Ag-loadings.

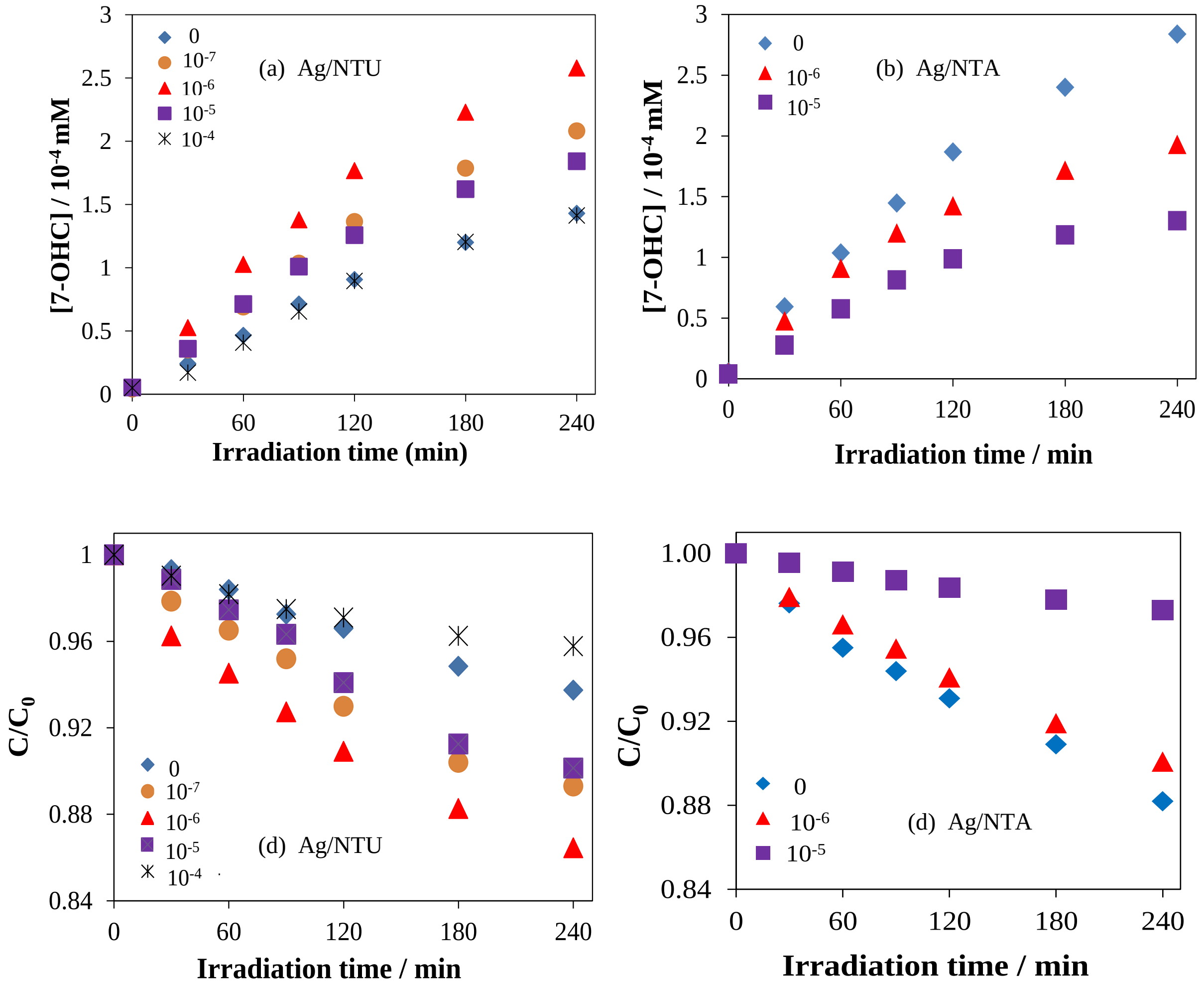

Figure S5. (a-b) 7-OHC formation and (c-d) coumarin degradation over Ag/NTU and Ag/NT-A with different Ag-loadings.

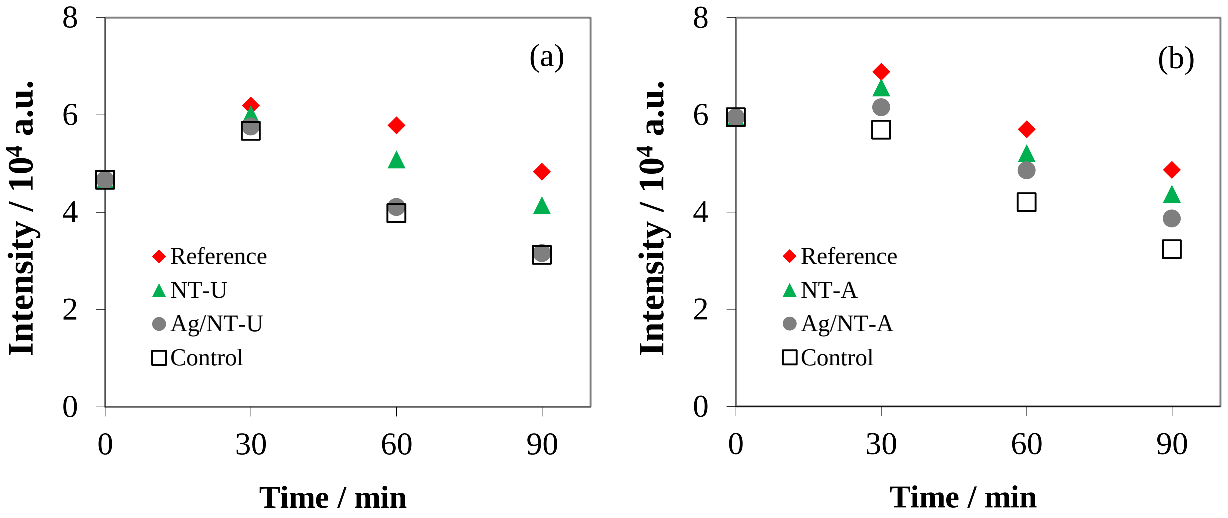

Figure S6. Luminescence intensity of bacterial suspension in the presence of (a) Ag/NT-U and (b) Ag/NT-A catalysts (with 10^{-6} mol g^{-1} Ag concentration) compared to the reference and control samples.

Table S2. Relative decomposition of bacteria in presence of NT-U and Ag/NT-U (with 10^{-6} mol g^{-1} Ag concentration) catalysts as well as the control sample.

Time / min	NT-U	Ag/NT-U	Control
0	0.00%	0.00%	0.00%
30	2.89%	7.01%	8.43%
60	12.06%	29.07%	31.25%
90	14.27%	34.64%	35.35%

Table S3. Relative decomposition of bacteria in presence of NT-A and Ag/NT-A (with 10^{-6} mol g^{-1} Ag concentration) catalysts as well as the control sample.

Time / min	NT-A	Ag/NT-A	Control
0	0.00%	0.00%	0.00%
30	4.62%	10.61%	17.32%
60	8.63%	14.83%	26.32%
90	10.09%	20.58%	33.66%
